# Dangerous space emphysema after dental treatment

**DOI:** 10.4103/1817-1737.65041

**Published:** 2010

**Authors:** Abdulrahman Hagr

**Affiliations:** King Abdulaziz University, King Saud University, Hospital Riyadh, Saudi Arabia

**Keywords:** Dangerous space, dental abscess, emphysema

## Abstract

We report the case of an elderly female patient who presented with dangerous space emphysema occurring after a dental procedure. This case presented a diagnostic and management dilemma because of the development of an unusual complication of dental disease. In our review of the medical literature, we were unable to find any cases with similar manifestations and clinical courses.

Emphysema of dangerous space is a rare complication of dental procedures. Although this disease has potentially fatal complications such as vascular injuries and mediastinitis, patients can present with minimal signs and symptoms. We report a case of emphysema of dangerous space discovered after tooth extraction and drainage of a submandibular abscess.

## Case Report

A 69-year-old female, with a long history of poorly controlled diabetes and hypertension, presented to the emergency room with a one month history of toothaches that were treated conservatively by her dentist using broad spectrum antibiotics. In the emergency room, she was drowsy and lethargic but not disoriented, with a temperature of 38.6°C. Her pulse was 112, and her blood pressure was 136/79. She had submandibular swelling with complete trismus. On admission, she had leukocytosis (16 000) and normal chest radiographs. She underwent exploration under general anesthesia using nasotracheal intubation. Periapical infection of tooth 48 and a submandibular abscess were found. A maxillofacial surgeon extracted tooth 48 and incised and drained the submandibular dental abscess. Hydrogen peroxide was used intraoperatively and postoperatively through the drain. The patient showed marked improvement regarding her trismus post-op. However, she developed progressively more severe dysphagia post-op. Since she did not improve after two days, ENT and GI services were consulted.

**Figure 1:**
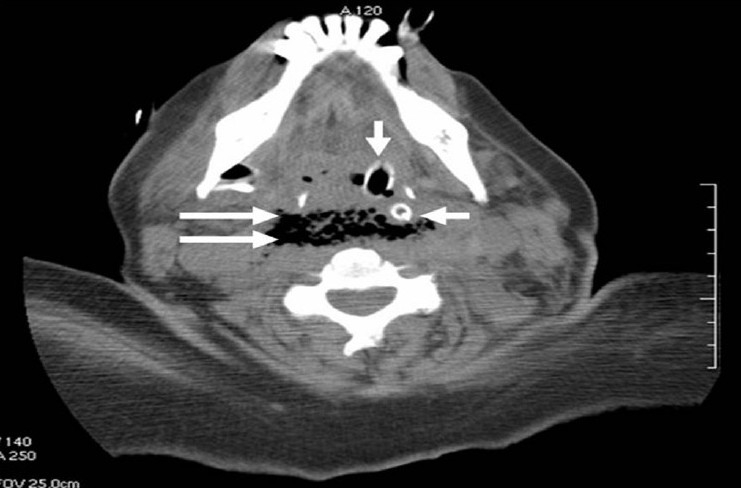
An axial computed tomogram (non-enhanced) reveals free air and air bubbles within the dangerous space (two horizontal arrows), ET tube (vertical arrow) and NG tube (one horizontal arrow)

We found that the patient had edema of the soft palate, deviation of the right tonsil to the midline and laceration in the posterior wall of the oropharynx. Because the patient had high creatinine levels at that time, neck computed tomography (CT) without contrast was performed. The CT showed massive soft tissue widening in the parapharyngeal area and dangerous space with free air and air bubbles [Figure 1]. This free air extended from the nasopharynx down to the mediastinum. The gastroenterologist performed gastroscopy and endoscopy, finding a duodenal ulcer, but nothing anatomical obstructing the upper gastrointestinal tract.

The patient was placed on amikacin, clindamycin and ceftazidime. The intraoperative culture was negative, but later *Escherichia coli* was cultured from the pus exuding from the drain and on the dressings. These *E. coli* were resistant to all antibiotics except ceftazidime. The patient was put on metronidazole and ceftazidime and showed some improvement. The maxillofacial surgeons decided to explore and clean the parapharyngeal space under general anesthesia. Intraoperatively, the patient had difficulty with the orotracheal intubation due to narrowing of the airway and copious amount of pus in the pharynx. The parapharyngeal space was drained externally and vigorous irrigation with hydrogen peroxide was performed. Postoperatively, the patient was admitted to the ICU and was found to have S–T segment elevation and multiple PVCs, which the cardiologist diagnosed as MI with LBBB. The echocardiogram showed concentric LVH with mild diastolic dysfunction as well as degenerative valvular disease (mild mitral, aortic and tricuspid regurgitation). Moreover, liver tests showed low albumin levels and elevated enzyme levels. The patient was kept intubated in the ICU, and copious amount of pus was suctioned through the mouth, but nothing drained through the parapharyngeal space drain. Her chest radiograph revealed a bilateral infiltrate suggesting aspiration pneumonia and bilateral pleural effusion.

**Figure 2:**
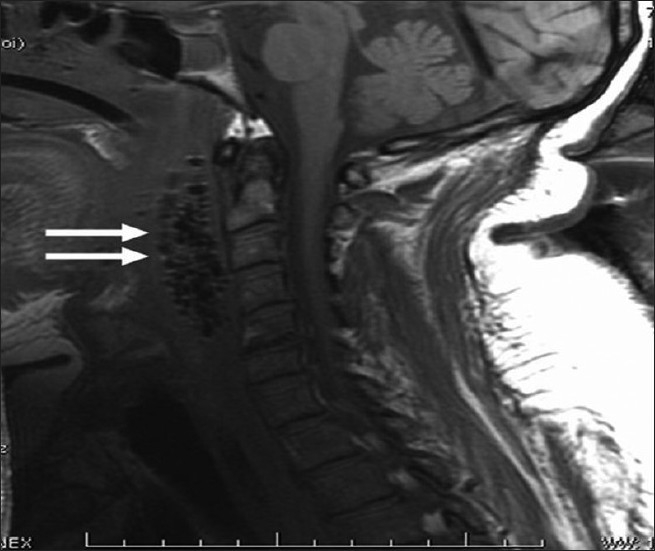
A sagittal T1 cervical MRI reveals air and air-bubble accumulation in the dangerous space (two arrows)

We decided to evaluate the retropharyngeal area using MRI to exclude any neck collection. The MRI was also performed without contrast due to high creatinine levels, and it did not show any neck collection. However, air bubbles were found in the dangerous space [Figure 2]. The patient underwent tracheotomy and exploration of the dangerous space. Intraoperatively, the patient was found to have ulceration of the oropharyngeal wall with necrotic tissue in the sub-mucosa. External neck incision was performed, and the dangerous space was connected to the rest of the neck spaces. Thorough debridement of necrotic tissue was completed, followed by irrigation with hydrogen peroxide. A through-and-through drain was used at the end of surgery to irrigate and drain collection in the dangerous space postoperatively. Oral feeding was stopped, a nasogastric feeding tube was inserted and enteral feeding was started. The patient showed marked improvement post-op.

A week later, we performed suspension pharyngoscopy, which showed marked improvement of the posterior wall of the oro-pharynx and disappearance of the necrotic tissue. We repaired the pharyngeal opening of the fistulae with absorbable sutures and kept the drain in the external orifices to avoid re-accumulation of air and pus. The patient restarted with feeding and was discharged soon afterwards.

## Discussion

The spread of large amounts of air into deep neck spaces may sometimes cause serious complications, including cardiac and respiratory failure from pneumopericardium, pneumomediastinum, or airway compromise due to the accumulation of air in the retropharyngeal space. Moreover, fatal air embolism and soft tissue infections can occur through dissemination of oral microflora along the emphysematous aero-digestive tracts.[1] If this condition is not diagnosed early and adequately treated, the patient may develop a life-threatening upper airway obstruction.[2]

Our patient is the fourth reported pneumothorax case associated with dental treatment published to date.[3–5] The development of deep soft tissue cervicofacial emphysema after dental treatment is a rare complication,[3,6] and most patients who develop subcutaneous emphysema after a dental procedure have only moderate local swelling.[3] In these cases, air dissection is probably caused by pressurized air being forced through the disrupted intraoral barrier (dentoalveolar membrane or root canal).[6] The bases of the first, second and third molars directly communicate with the sublingual and submandibular spaces. These spaces, in turn, communicate with the parapharyngeal, which communicates with all deep neck spaces, including the dangerous space. Therefore, any dental procedure in this area can lead to deep neck emphysema.

In conclusion, early and accurate diagnosis of air in the dangerous space is very important to avoid potential complications. The severe odynophagia and dysphagia are not commonly recognized initial features of air in the dangerous space of the neck due to dental procedures. Free air in the dangerous space could extend downward into the mediastinum and upward intracranially via fascial planes. The diagnosis is best established radiologically.
